# Dancing Towards Stability: The Therapeutic Potential of Argentine Tango for Balance and Mobility in Parkinson’s Disease

**DOI:** 10.3390/diseases13030082

**Published:** 2025-03-13

**Authors:** Federica Giorgi, Daniela Platano, Lisa Berti, Danilo Donati, Roberto Tedeschi

**Affiliations:** 1 Pediatric Physical Medicine and Rehabilitation Unit, IRCCS Institute of Neurological Sciences, 40126 Bologna, Italy; federica.giorgi15@gmail.com; 2 Department of Biomedical and Neuromotor Sciences, Alma Mater Studiorum, University of Bologna, 40126 Bologna, Italy; daniela.platano@unibo.it (D.P.); lisa.berti@unibo.it (L.B.); 3 Physical Medicine and Rehabilitation Unit, IRCCS Istituto Ortopedico Rizzoli, 40136 Bologna, Italy; 4 Physical Therapy and Rehabilitation Unit, Policlinico di Modena, 41125 Modena, Italy; danilo.donati@unimore.it; 5 Clinical and Experimental Medicine PhD Program, University of Modena and Reggio Emilia, 41125 Modena, Italy

**Keywords:** Parkinson’s disease, Argentine Tango, balance rehabilitation, gait improvement, non-pharmacological therapy

## Abstract

**Background:** Parkinson’s Disease (PD) is a progressive neurodegenerative disorder characterized by motor impairments, including balance deficits, gait disturbances, and postural instability. Given the limitations of pharmacological treatments, alternative rehabilitative strategies such as Argentine Tango (AT) have been explored for their potential benefits in improving mobility and quality of life in individuals with PD. This systematic review evaluates the effectiveness of AT in enhancing balance, gait, and functional mobility in PD patients. **Methods:** A systematic literature search was conducted across PubMed, Cochrane Central Register of Controlled Trials (CENTRAL), Scopus, PEDro, and Web of Science. Studies were included if they were randomized controlled trials (RCTs) assessing the impact of AT on motor outcomes in PD. Data extraction and risk of bias assessment were performed independently by two reviewers using the Risk of Bias 2 (RoB 2) tool. **Results:** Five randomized controlled trials (RCTs) were included, with sample sizes ranging from 10 to 62 participants and intervention durations varying from 10 weeks to 24 months. AT significantly improved balance (Mini-BESTest, BBS, FRT), gait performance (6MWT, TUG), and mobility compared to usual care or conventional exercise. Some studies also reported psychological benefits, including reduced depressive symptoms and increased balance confidence. However, freezing of gait outcomes were inconclusive, and methodological limitations, such as small sample sizes and inconsistent intervention durations, were noted. Outcomes for freezing of gait (FoG) remained inconclusive due to the variability in assessment methods and inconsistent reporting across studies. **Conclusions:** AT appears to be an effective rehabilitation strategy for improving balance, gait, and functional mobility in PD. While preliminary evidence suggests additional psychological benefits, larger, high-quality trials are needed to confirm its long-term efficacy and establish standardized intervention protocols. AT may be integrated into multimodal rehabilitation programs to enhance motor and psychosocial outcomes in PD management. However, the small sample sizes of included studies and the heterogeneity in intervention durations limit the generalizability of findings. AT may serve as a structured rehabilitative approach for improving mobility and psychosocial outcomes in PD and could be integrated into community-based or clinical rehabilitation programs.

## 1. Introduction

Parkinson’s Disease (PD) is a progressive neurodegenerative disorder characterized by the degeneration of dopaminergic neurons, leading to hallmark motor symptoms such as bradykinesia, rigidity, resting tremor, and postural instability [1,2,3,4,5,6,7,8]. In addition to motor impairments, PD is associated with non-motor symptoms, including cognitive decline, mood disturbances, and autonomic dysfunction, which significantly impact patients’ quality of life and contribute to increased disability [9,10,11,12]. The prevalence of PD is rising globally, with projections indicating a substantial increase in affected individuals in the coming decades. Given the chronic and progressive nature of PD, long-term management strategies that extend beyond pharmacological treatments are increasingly emphasized in clinical research and practice [13,14,15,16,17]. Various dance-based therapies, including ballroom, contemporary, and folk dance, have been explored as rehabilitation strategies in PD. These interventions aim to improve motor function, cognitive engagement, and emotional well-being, with some evidence suggesting benefits comparable to structured exercise programs. Current pharmacological treatments, including dopaminergic medication and deep brain stimulation, provide symptomatic relief but do not halt disease progression [4,10,18,19]. These treatments are also associated with limitations, such as motor fluctuations and dyskinesias. As a result, non-pharmacological interventions have gained attention for their potential to improve motor function, cognitive performance, and overall well-being in individuals with PD [20,21,22,23,24,25]. Among these, dance-based therapies have emerged as particularly promising, integrating physical exercise, cognitive engagement, and social interaction into a single therapeutic approach. Argentine Tango (AT) has been proposed as a novel rehabilitative strategy for individuals with PD [26,27,28,29,30,31,32,33,34,35]. AT requires complex motor sequences, demanding focus on rhythmic movements, postural adjustments, and partner coordination. This combination of structured yet adaptive movement patterns makes AT an appealing approach for addressing key motor deficits in PD, particularly balance impairments and gait dysfunction [17,25,36,37,38]. Additionally, AT incorporates external cues (such as music and partner feedback), which have been shown to facilitate movement initiation and fluidity in individuals with PD. AT offers unique motor and cognitive demands, requiring continuous adaptation to partner-led movements, external rhythmic cues, and multidirectional stepping patterns. Unlike other dance forms, AT emphasizes postural control, weight shifting, and improvisation, which may offer enhanced benefits for PD-specific gait and balance deficits. Given its dual emphasis on mobility and engagement, AT is hypothesized to improve balance, reduce freezing of gait, and enhance walking ability in this population. Despite increasing interest in AT as a rehabilitative intervention, inconsistencies remain in the literature regarding its efficacy. Despite increasing interest in AT as a rehabilitative intervention, inconsistencies remain in the literature regarding its efficacy [26]. Variability in study methodologies, intervention durations, and outcome measures has led to conflicting results. While several randomized controlled trials (RCTs) report significant improvements in balance and mobility among individuals with PD following AT training, other studies present mixed or inconclusive findings [26,28,31,39,40]. The variability in study designs, intervention durations, outcome measures, and comparison groups makes the interpretation of findings more challenging, emphasizing the need for a systematic synthesis of the available evidence. This systematic review evaluates the effectiveness of AT in enhancing balance, gait, functional mobility, and psychological outcomes in PD patients. This systematic review aims to evaluate the effectiveness of AT as a rehabilitative intervention for individuals with PD. Specifically, this review seeks to assess the impact of AT on key motor outcomes, including balance and walking performance, compare AT with conventional rehabilitation approaches to determine its relative effectiveness, and identify methodological limitations and gaps within the existing research to guide future investigations. By systematically consolidating findings from multiple studies, this review intends to provide a comprehensive understanding of AT’s rehabilitative potential and inform clinical recommendations for its integration into non-pharmacological management strategies for PD.

## 2. Methods

The present systematic review was conducted in accordance with the methodological framework established by the Joanna Briggs Institute (JBI) [41], specifically designed for systematic reviews. To maintain a high level of rigor and transparency, the review process followed the guidelines set forth in the PRISMA Statement for Systematic Reviews (PRISMA) [42]. This systematic review protocol was preregistered on the Open Science Framework (OSF) at https://osf.io/fwgcm accessed on 1 December 2024.

### 2.1. Review Question

We formulated the following research question: “ What is the effectiveness of Argentine Tango as a rehabilitative intervention for individuals with Parkinson’s Disease in improving balance and walking performance compared to conventional rehabilitation approaches?”

### 2.2. Eligibility Criteria

Studies were eligible for inclusion if they met the following Population, Concept, and Context (PCC) criteria.

Population (P): This review includes studies involving individuals diagnosed with Parkinson’s Disease (PD), regardless of disease stage, age, sex, or duration of the condition. Participants may present with varying levels of motor impairment, including but not limited to bradykinesia, postural instability, and gait dysfunction. Studies focusing on individuals with atypical parkinsonian syndromes (e.g., multiple system atrophy or progressive supranuclear palsy) are excluded unless explicitly distinguished from idiopathic PD.

Concept (C): The primary concept of interest is the effectiveness of Argentine Tango (AT) as a rehabilitative intervention for individuals with PD. This includes assessing AT’s impact on motor functions such as balance, gait performance, and freezing of gait, as well as secondary outcomes like quality of life, fall risk, cognitive function, and psychosocial well-being. Studies employing AT as the primary intervention, either alone or in combination with standard rehabilitation approaches, are considered. Studies that investigate other dance-based interventions without isolating AT’s effects are excluded.

Context (C): Eligible studies must evaluate AT interventions in a rehabilitation or therapeutic setting, including clinical, community, or research environments where AT is delivered in structured sessions with professional guidance. Studies set in purely recreational or competitive dance settings without an explicit rehabilitative aim are excluded. The review considers studies conducted globally, without geographic restrictions, as long as they provide sufficient methodological rigor and report outcomes relevant to PD rehabilitation.

### 2.3. Exclusion Criteria

Studies evaluating general dance therapies were excluded to maintain methodological homogeneity. AT was selected due to its structured yet adaptive nature, which incorporates specific postural and rhythmic components that directly target PD-related motor impairments.

### 2.4. Search Strategy

A systematic search was initially conducted in MEDLINE via PubMed to identify relevant studies. The keywords and indexing terms retrieved from these preliminary results were then used to refine a comprehensive search strategy. This approach was subsequently adapted and implemented across multiple databases, including the Cochrane Central Register of Controlled Trials (CENTRAL), Scopus, PEDro, and Web of Science, ensuring extensive coverage of the literature. The database searches were completed on 23 January 2024, with no restrictions on publication date. Below are the detailed search strategies tailored for each database:

MEDLINE(PubMed):

(“Parkinson Disease”[Mesh] OR “Parkinson’s Disease” OR “PD” OR “Idiopathic Parkinson’s Disease”) AND (“Tango” OR “Argentine Tango” OR “Dance Therapy”) AND (“Balance”[Mesh] OR “Gait”[Mesh] OR “Postural Control” OR “Mobility” OR “Walking Speed” OR “Locomotion”) AND (randomized controlled trial[Publication Type] OR “RCT” OR “Clinical Trial” OR “Controlled Clinical Trial” OR “Trial” OR “Intervention Study”)

Cochrane Central:

(“Parkinson Disease” OR “Parkinson’s Disease” OR “Idiopathic Parkinson’s Disease”) AND (“Argentine Tango” OR “Tango” OR “Dance-Based Therapy”) AND (“Balance” OR “Postural Stability” OR “Gait” OR “Mobility” OR “Walking Speed”) AND (RCT OR “Randomized Controlled Trial” OR “Clinical Trial”)

Scopus:

TITLE-ABS-KEY(“Parkinson Disease” OR “Parkinson’s Disease” OR “Idiopathic Parkinson’s Disease”) AND TITLE-ABS-KEY(“Argentine Tango” OR “Tango” OR “Dance Therapy”) AND TITLE-ABS-KEY(“Balance” OR “Postural Stability” OR “Gait” OR “Mobility” OR “Walking Speed”) AND (LIMIT-TO(DOCTYPE, “ar”) OR LIMIT-TO(DOCTYPE, “cp”))

PEDro:

Parkinson AND Tango AND (Balance OR Gait OR Mobility OR Walking Speed)

Web of Science:

TS=(“Parkinson Disease” OR “Parkinson’s Disease” OR “Idiopathic Parkinson’s Disease”) AND TS=(“Argentine Tango” OR “Tango” OR “Dance Therapy”) AND TS=(“Balance” OR “Postural Stability” OR “Gait” OR “Mobility” OR “Walking Speed”) AND TS=(“Randomized Controlled Trial” OR “RCT” OR “Intervention Study”)

### 2.5. Study Selection

The study selection process was carried out using a structured and methodical approach in accordance with scoping review guidelines. Following the search, all results were compiled, and duplicate entries were systematically removed using Zotero. The screening was conducted in two sequential phases: first, an initial review of titles and abstracts was performed to exclude irrelevant studies, followed by a detailed full-text assessment of the remaining eligible articles. Both stages of the screening process were independently undertaken by two reviewers, with a third reviewer providing adjudication in cases of disagreement. To uphold transparency and methodological rigor, the entire selection process adhered to the PRISMA 2020 guidelines. Two independent reviewers screened the titles and abstracts of all retrieved studies. Full-text articles were then assessed for eligibility according to the predefined inclusion criteria. In cases of disagreement, a third senior reviewer was consulted to reach a consensus. To quantify inter-rater reliability during the screening phase, we calculated Cohen’s kappa (κ) coefficient, which yielded a κ value of 0.70, indicating substantial agreement.

### 2.6. Data Extraction and Data Synthesis

**Figure 1:**
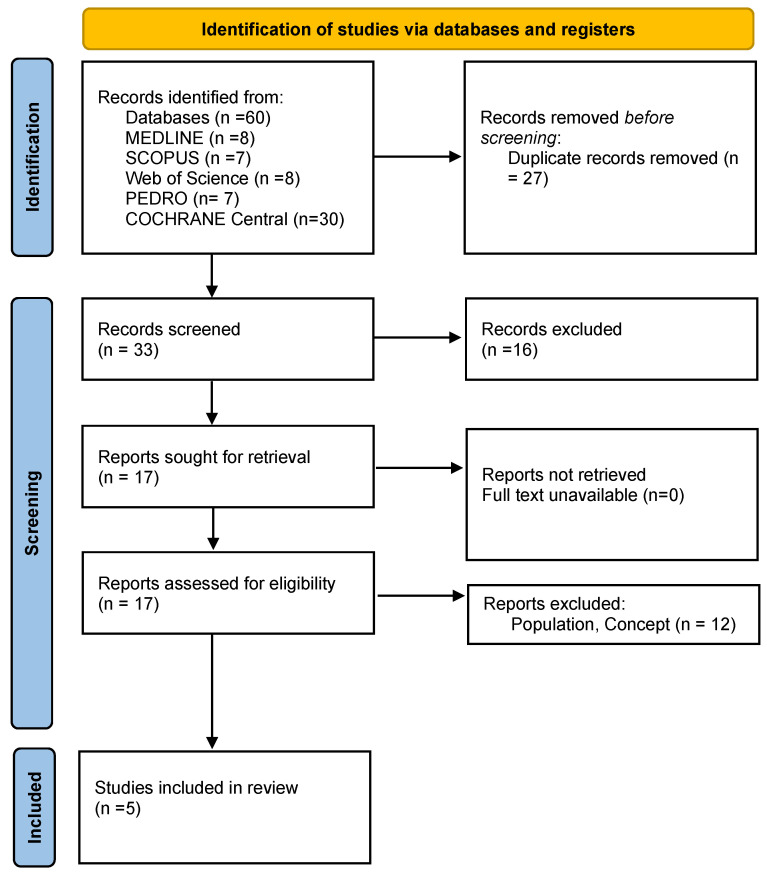
Preferred reporting items for systematic reviews and meta-analyses 2020 (PRISMA) flow-diagram.

Data extraction was systematically performed to gather essential information, including study design, participant characteristics, intervention specifics, reported outcomes, and key findings. A standardized data extraction form was employed to ensure uniformity across studies and maintain consistency in data collection. Outcomes were systematically categorized to facilitate comparative analysis, with qualitative assessment used to identify emerging patterns and research gaps. Additionally, quantitative data were synthesized to highlight significant trends and key results. This structured approach enabled a comprehensive and methodical synthesis, aligning with the research objectives. Conflicting results across studies were addressed by considering study quality, intervention parameters, and outcome measure consistency. A narrative synthesis approach was employed to identify patterns and discrepancies across included studies. This review was conducted following the Preferred Reporting Items for Systematic Reviews and Meta-Analyses (PRISMA) guidelines. A PRISMA flow diagram (Figure 1) was used to depict the study selection process, and a PRISMA checklist was followed to ensure transparency and methodological rigor. A formal statistical heterogeneity analysis (e.g., I^2^ statistic) was not conducted due to the small number of included studies (n = 5) and the variability in intervention durations, outcome measures, and study designs. However, a qualitative assessment of heterogeneity was performed by comparing differences in study populations, intervention protocols, and outcome reporting. This variability was considered when interpreting results and drawing conclusions.

## 3. Results

**Figure 2:**
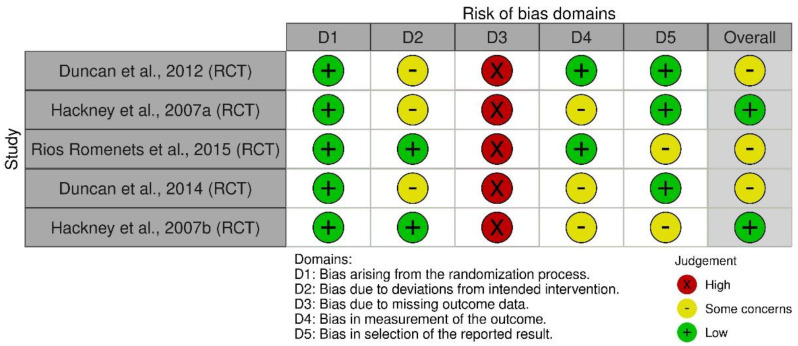
Risk of Bias Assessment for Included Studies [29,30,31,32,33].

As presented in the PRISMA 2020 flow diagram (Figure 1), the initial literature search identified 60 records. After screening, 55 studies were excluded, resulting in the inclusion of 5 articles (Table 1). The quality assessment of these studies, conducted using the Risk of Bias 2 (RoB 2) tool, is presented in Figure 2, providing an overview of potential methodological limitations across different bias domains.

### 3.1. Balance and Postural Stability

All studies included in this review assessed balance using various standardized tools. Duncan et al. (2012) [29] and Rios Romenets et al. (2015) [31] demonstrated significant improvements in balance after Argentine Tango (AT) training, with the Mini-BESTest showing marked increases in postural stability. Hackney et al. (2007a) [30] reported greater improvements in the Berg Balance Scale (BBS) scores in the AT group compared to a traditional exercise group, suggesting superior benefits for dynamic balance. Similarly, Hackney et al. (2007b) [33] noted that PD participants in the AT group experienced enhanced postural control and improved functional reach as measured by the Functional Reach Test (FRT) and One Leg Stance Test (OLS). Duncan et al. (2014) [32] further indicated that participants who underwent AT training for 24 months were able to maintain balance and postural stability, while those in the control group exhibited a decline over time.

### 3.2. Gait and Mobility

Gait performance was assessed through measures such as the Six-Minute Walk Test (6MWT) and the Timed Up and Go (TUG). Duncan et al. (2012) [29] and Duncan et al. (2014) [32] both reported significant increases in walking distance in the AT group, highlighting improved endurance and mobility. The 6MWT scores were notably higher in participants engaged in at, while TUG times were reduced, indicating better movement efficiency. Rios Romenets et al. (2015) [31] found that at participants demonstrated enhanced walking speed and postural adaptability. Hackney et al. (2007a) [30] also observed that the AT group showed improvements in gait performance metrics, but without significant changes in Unified Parkinson’s Disease Rating Scale (UPDRS) scores related to motor symptoms.

### 3.3. Freezing of Gait

The effectiveness of AT in reducing freezing of gait (FoG) was inconsistent across studies. Duncan et al. (2012) [29] reported no significant improvement in FoG episodes despite enhanced gait performance. Rios Romenets et al. (2015) [31] did not specifically assess FoG but noted overall improvements in walking adaptability. Hackney et al. (2007b) [33] observed that participants in the AT group exhibited greater balance confidence, which may indirectly contribute to reduced freezing episodes, but this was not directly measured.

### 3.4. Functional Mobility and Long-Term Effects

Duncan et al. (2014) [32] provided valuable insight into the long-term impact of AT, showing that participants who engaged in 24 months of AT maintained functional mobility levels, whereas the control group exhibited declines. This suggests that AT may serve as a viable long-term rehabilitation strategy for individuals with PD, helping to preserve motor function and prevent deterioration.

### 3.5. Cognitive and Psychological Outcomes

Some studies incorporated cognitive and psychological assessments to evaluate the broader effects of AT. Rios Romenets et al. (2015) [31] found that AT training led to significant reductions in depressive symptoms, as measured by the Beck Depression Inventory (BDI), whereas cognitive improvements assessed using the Montreal Cognitive Assessment (MoCA) were not significant. Hackney et al. (2007b) [33] reported increased balance confidence in AT participants, measured via the Activities-specific Balance Confidence (ABC) scale, suggesting a positive impact on perceived mobility and self-efficacy.

This figure presents the Risk of Bias (RoB 2) assessment for the included randomized controlled trials evaluating the effects of Argentine Tango on Parkinson’s Disease rehabilitation. The RoB 2 tool is a standardized framework for evaluating methodological quality in RCTs, assessing bias domains such as randomization process, deviations from intended interventions, missing outcome data, measurement bias, and selective reporting bias. Across studies, selection bias was minimized through randomization; however, the performance bias was high due to the impossibility of blinding participants in dance interventions. Reporting bias was low, but small sample sizes contributed to potential detection bias in outcome measures.

Adherence rates to AT interventions varied, with studies reporting dropout rates ranging from 12% to 30%. Factors influencing adherence included session frequency, accessibility of dance training, and individual motivation. Future studies should explore the feasibility of long-term AT programs and their impact on sustained motor benefits.

## 4. Discussion

The results of this systematic review indicate that Argentine Tango (AT) is a promising rehabilitative approach for individuals with Parkinson’s Disease (PD), particularly for improving balance, gait performance, and functional mobility. Across the five randomized controlled trials (RCTs) included, participants who engaged in AT training showed significant improvements in balance measures, such as the Mini Balance Evaluation Systems Test (Mini-BESTest) [43], Berg Balance Scale (BBS) [44], and Functional Reach Test (FRT) [45]. These findings are particularly relevant, given that balance impairment is one of the major contributors to falls and reduced mobility in individuals with PD, often leading to increased disability and loss of independence.

A key factor in the observed balance improvements may be the structured, yet adaptable, nature of AT. The dance requires precise postural adjustments, weight shifting, and multi-directional stepping patterns, all of which contribute to enhanced dynamic stability. Furthermore, the reliance on external cues, such as music and partner feedback, may facilitate movement initiation and postural control, making AT particularly effective for individuals experiencing bradykinesia or difficulty with motor planning. This aligns with previous research suggesting that external rhythmic cues can help bypass basal ganglia deficits and enhance motor execution in PD patients. While AT has demonstrated benefits in balance and gait, other non-pharmacological interventions, such as tai chi and virtual reality-based rehabilitation, have shown comparable effects. Further comparative studies are needed to delineate the relative advantages of each approach.

The observed motor improvements following Argentine Tango (AT) interventions can be explained by several neurophysiological mechanisms. AT integrates multiple rehabilitation principles, including external rhythmic cueing, proprioceptive feedback, postural control strategies, and cognitive-motor integration. External auditory and visual cues provided through music and partner coordination may enhance basal ganglia-thalamocortical circuit activation, facilitating motor initiation and movement fluidity. Additionally, the requirement for precise weight shifting and adaptive stepping patterns engages balance mechanisms, potentially improving postural stability and reducing freezing episodes. The cognitive demand of AT, which requires continuous motor planning and execution, may further contribute to gait improvements through enhanced attentional control. These findings align with previous research on rhythmic-based interventions in Parkinson’s Disease (PD), highlighting the role of structured movement therapies in modulating motor and cognitive function. Future studies should investigate whether these mechanisms are maintained long-term and how they compare with other non-pharmacological interventions.

In addition to balance, gait performance and mobility were substantially improved in AT participants. Several studies, including Duncan et al. (2012) [29] and Duncan et al. (2014) [32], reported significant increases in walking distance (measured by the Six-Minute Walk Test, 6MWT) and reductions in Timed Up and Go (TUG) test times, indicating that AT contributes to better endurance, walking efficiency, and postural transitions. These results support the notion that dance-based interventions encourage more natural and fluid movement patterns, potentially mitigating the rigidity and festination often observed in PD gait disturbances. External rhythmic cues engage the basal ganglia-thalamocortical circuitry, facilitating motor initiation and reducing bradykinesia. This aligns with evidence suggesting that auditory cueing enhances sensorimotor integration in PD.

However, the effect of AT on freezing of gait (FoG) remains inconclusive. While some studies noted improved gait fluidity and reduced hesitation during movement transitions, there was no direct evidence indicating a reduction in the frequency or severity of freezing episodes. This is a critical area for further investigation, as FoG significantly affects quality of life and fall risk in PD. Future research should aim to establish whether AT directly influences the neural circuits involved in FoG or whether its benefits are primarily limited to general gait and postural stability improvements. Future studies should implement standardized FoG assessment tools, such as the Freezing of Gait Questionnaire (FOG-Q) and spatiotemporal gait analysis, to improve consistency across trials.

Another important outcome highlighted in this review is the psychosocial and psychological impact of AT. Rios Romenets et al. (2015) [31] reported a significant reduction in depressive symptoms among AT participants, as assessed by the Beck Depression Inventory (BDI). Additionally, Hackney et al. (2007b) [33] found that AT led to improved balance confidence, measured using the Activities-specific Balance Confidence (ABC) scale. These findings align with broader literature indicating that dance therapy may have cognitive and emotional benefits beyond its motor effects. The social interaction and engagement required in AT may contribute to enhanced mood, motivation, and adherence to rehabilitation programs, making it an appealing option for long-term therapeutic interventions. Argentine Tango also facilitates social interaction, which is particularly relevant for PD patients, who often face isolation. The structured partner-based format of AT may promote social engagement, potentially contributing to improved mood and adherence to rehabilitation programs. The integration of cognitive, sensorimotor, and social components in AT likely contributes to its therapeutic effects. Dance-induced neuroplasticity may enhance proprioceptive feedback and movement automatization, further supporting motor improvements.

Despite these promising findings, the quality of the included studies varied, as reflected in the Risk of Bias 2 (RoB 2) assessment (Figure 2). While random sequence generation was well-reported in most studies, allocation concealment was often unclear, introducing some concerns regarding potential selection bias. Additionally, blinding of participants and personnel was not feasible due to the nature of AT, leading to high risk of bias in this domain. This is a common limitation in rehabilitation research, where participant engagement is an essential component of the intervention. To mitigate this issue in future studies, researchers should consider blinding outcome assessors and using objective biomechanical assessments where possible.

## 5. Limitations

Several limitations must be considered when interpreting the results of this review. The majority of included studies had relatively small sample sizes, ranging from 10 to 62 participants, which may limit the generalizability of findings. Larger, multi-center trials are needed to confirm the efficacy of AT across diverse PD populations. Additionally, the reviewed studies utilized different AT training durations, ranging from 10 weeks to 24 months, making it difficult to determine the optimal dose–response relationship. Standardized protocols should be developed to establish the minimum required training duration for meaningful clinical improvements. While some studies measured mood and balance confidence, there was a limited inclusion of cognitive outcomes. Given the potential for dance-based interventions to influence executive function, attention, and visuospatial abilities, future research should incorporate neuropsychological assessments and patient-reported outcome measures to capture the full scope of benefits. Moreover, while AT was compared to traditional exercise or usual care, none of the studies directly compared AT to other dance-based interventions, such as ballroom or contemporary dance. Future trials should explore whether AT provides unique advantages over alternative dance therapies. Another important consideration is long-term sustainability and adherence. While Duncan et al. (2014) [32] showed that AT maintained motor improvements over 24 months, few studies examined adherence beyond the trial period. Future research should investigate whether participants continue practicing AT independently and how this affects long-term outcomes. The findings are limited by small sample sizes and variable intervention durations. Larger, multi-center trials with extended follow-up periods are needed to establish long-term efficacy.

### Clinical Practice Implications

The findings of this review suggest that Argentine Tango could be effectively integrated into rehabilitation programs for individuals with PD, particularly for those experiencing balance and gait impairments. Several practical considerations should be taken into account for clinical implementation. Given the complexity of AT movements, programs should be conducted by therapists trained in both dance and movement disorders to ensure safe and effective instruction. While AT was beneficial for Hoehn and Yahr stage 2–3 patients, its feasibility for advanced PD cases remains unclear, and modified dance strategies and adaptive techniques should be explored for patients with greater mobility restrictions. The rhythmic and partner-based nature of AT provides real-time feedback and auditory cues, which may enhance motor learning and movement initiation. AT should be considered as part of a comprehensive rehabilitation program alongside physical therapy, strength training, and assistive technologies. Encouraging patients to continue AT beyond structured programs may help sustain mobility benefits and reduce fall risk over time. AT can be implemented through community-based programs, hospital outpatient rehabilitation settings, and tele-rehabilitation platforms. Training rehabilitation professionals in dance-based therapy can enhance accessibility and effectiveness.

## 6. Conclusions

This systematic review supports the use of Argentine Tango as a viable non-pharmacological intervention for Parkinson’s Disease rehabilitation, particularly for improving balance, gait, and functional mobility. Additionally, AT may provide psychological benefits such as reduced depressive symptoms and increased confidence in movement, further enhancing its value in clinical practice. However, larger, well-controlled trials are needed to refine intervention protocols, assess cognitive and long-term adherence outcomes, and compare AT with other rehabilitation strategies. Given its structured yet engaging format, AT represents a promising addition to current rehabilitation practices, offering both motor and psychosocial benefits for individuals with PD. Future research should explore the long-term effects of AT, its comparative efficacy against other dance therapies, and its integration into multimodal rehabilitation programs.

## Figures and Tables

**Table 1:** Summary of Included Studies.

Author, Year, and Study Type	Methods	Results	Outcomes Achieved	Level of Evidence
Duncan et al., 2012 (RCT) [29]	Sixty-two participants with PD (mean age: 69 years, Hoehn and Yahr stage 2–3), intervention group received 12-month AT training (2 sessions/week), control group continued usual physical activity. Balance and gait assessed using Mini-BESTest, 6MWT, and UPDRS-III.	AT group showed significant improvements in balance and gait at 6 and 12 months compared to controls.	Significant improvement in Mini-BESTest scores, 6MWT distance, and gait speed. No significant changes in freezing of gait.	Moderate (RCT, n = 62, long duration)
Hackney et al., 2007a (RCT) [30]	Nineteen PD patients (mean age: 72 years, Hoehn and Yahr stage 2–3), randomized to AT or traditional exercise. Intervention: 20 sessions over 10 weeks. Outcomes assessed via BBS, TUG, and UPDRS.	AT group demonstrated greater balance improvements (BBS) than the exercise group, but no significant differences in UPDRS scores.	Improved postural stability, dynamic balance, and gait performance. No significant motor symptom reduction.	Moderate (RCT, n = 19, short duration)
Rios Romenets et al., 2015 (RCT) [31]	Thirty-three participants with PD (mean age: 67 years, Hoehn and Yahr stage 2–3), randomized to AT or home-based exercise. Intervention: 12-week program (2 sessions/week). Evaluations included Mini-BESTest, TUG, MoCA, and BDI.	AT group showed greater improvements in balance (Mini-BESTest) and reduced depressive symptoms (BDI) compared to controls.	Enhanced postural stability, improved mood, no significant impact on cognitive function.	Moderate (RCT, n = 33, mid duration)
Duncan et al., 2014 (RCT) [32]	Ten PD patients (mean age: 70 years, Hoehn and Yahr stage 2–3) randomized to 24-month AT or usual physical activity. Outcomes assessed at 12 and 24 months via UPDRS, TUG, 6MWT.	AT group maintained balance and walking ability, while control group declined.	Long-term improvements in functional mobility, prevention of motor deterioration.	Low (RCT, n = 10, long duration)
Hackney et al., 2007b (RCT) [33]	Thirty-eight participants (19 PD, 19 healthy controls, mean age: 68 years, Hoehn and Yahr stage 2–3 for PD group), randomized to AT or conventional exercise. Balance and gait assessed via FRT, OLS, ABC scale.	PD participants in AT group reported improved balance confidence and postural control.	Increased functional reach, enhanced postural stability, improved perceived balance confidence.	Moderate (RCT, n = 38, mixed population)

Legend: ABC = Activities-specific Balance Confidence scale, AT = Argentine Tango, BBS = Berg Balance Scale, BDI = Beck Depression Inventory, FRT = Functional Reach Test, Mini-BESTest = Mini Balance Evaluation Systems Test, MoCA = Montreal Cognitive Assessment, OLS = One Leg Stance Test, PD = Parkinson’s Disease, RCT = Randomized Controlled Trial, TUG = Timed Up and Go, UPDRS = Unified Parkinson’s Disease Rating Scale, 6MWT = Six-Minute Walk Test.

## Data Availability

No new data were created or analyzed in this study. Data sharing is not applicable to this article.
